# DNMT1‐Induced Downregulation of CBX7 Inhibits ERK Phosphorylation and Promotes Pancreatic Ductal Adenocarcinoma Progression

**DOI:** 10.1096/fj.202402903R

**Published:** 2025-05-19

**Authors:** Haowei Shi, Xu Liu, Cheng Xing, Shiqi Guo, Yangyang Zheng, Wendan Tan, Yunpeng Ge, Jingyong Xu, Yao Li, Jinghai Song

**Affiliations:** ^1^ Department of General Surgery, Beijing Hospital, National Center of Gerontology, Institute of Geriatric Medicine Chinese Academy of Medical Sciences & Peking Union Medical College Beijing P. R. China; ^2^ National Cancer Center/National Clinical Research Center for Cancer/Cancer Hospital Chinese Academy of Medical Sciences and Peking Union Medical College Beijing P. R. China

**Keywords:** CBX7, DNMT1, pancreatic ductal adenocarcinoma, promoter methylation

## Abstract

Pancreatic ductal adenocarcinoma (PDAC) is one of the most aggressive cancer types, characterized by an alarmingly low 5‐year survival rate. DNA methylation has been implicated in the progression of various tumors, with DNA methyltransferase 1 (DNMT1) being the most extensively studied enzyme in this context. However, the expression patterns and underlying mechanisms of DNMT1 in PDAC remain poorly understood. The levels of DNMT1 and CBX7 in PDAC tissues and cells were determined by IHC and Western blot. ChIP and dual‐luciferase reporter assays confirmed the interaction between DNMT1 and the CBX7 promoter. Cellular functions were evaluated through CCK‐8, wound healing, and transwell assays. The expression of MAPK‐related proteins was analyzed by Western blot. DNMT1 expression was upregulated in PDAC tissues and cell lines, whereas CBX7 expression was downregulated. Silencing DNMT1 inhibited cell proliferation, migration, and invasion in PDAC by modulating CBX7 expression. Moreover, DNMT1 methylates the CBX7 promoter region, leading to increased ERK phosphorylation, which subsequently drives tumorigenesis and metastasis in PDAC. DNMT1 promotes the malignant progression of PDAC through the CBX7/ERK pathway. Our study provides evidence for potential therapeutic targets for the comprehensive treatment of PDAC.

## Graphical abstract

A schematic diagram illustrating the molecular mechanism by which DNMT1 regulates the CBX7/ERK axis in pancreatic ductal adenocarcinoma. DNMT1 may promote methylation of the CBX7 promoter, leading to the downregulation of CBX7 expression. This, in turn, enhances ERK phosphorylation, which inhibits the proliferation, migration, and invasion of pancreatic cancer cells, thereby contributing to the prevention of pancreatic ductal adenocarcinoma initiation and progression.

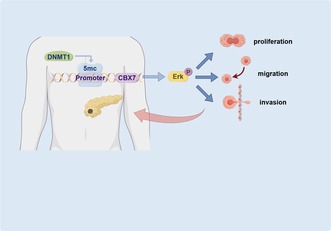

**Abbreviations**
CBX7: chromobox protein homolog 7
CCK‐8: cell counting kit‐8
ChIP: chromatin immunoprecipitation
DNMT1: DNA methyltransferase 1
ERK: extracellular regulated protein kinases
GSEA: gene set enrichment analysis
HPDE: human pancreatic duct epithelial
IHC: immunohistochemical assay
KEGG: kyoto encyclopedia of genes and genomes
MAPK: mitogen‐activated protein kinase
MS‐PCR: methylation‐specific PCR
PDAC: pancreatic ductal adenocarcinoma
PPI: Protein–Protein Interaction
PRC1: polycomb repressive complex 1
siRNA: small interfering RNA
TCGA: the Cancer Genome Atlas

## 1 Introduction

Pancreatic ductal adenocarcinoma (PDAC) is one of the most aggressive and lethal forms of cancer, accounting for more than 90% of all pancreatic cancer cases [1]. It is projected to become the second‐leading cause of cancer‐related deaths by 2030 in the United States [2]. Despite advancements in cancer research, PDAC continues to have a dismal prognosis, with a five‐year survival rate of less than 10% [3]. This poor survival rate is primarily due to the asymptomatic nature of the disease in its early stages, resulting in late diagnosis and its resistance to conventional therapies [4]. PDAC originates from the epithelial cells lining the pancreatic ducts and is characterized by rapid progression, extensive local invasion, and early metastasis [5, 6]. Given its complex biology and the challenges in early detection and treatment, PDAC remains a major unmet medical need, necessitating ongoing research into novel diagnostic and therapeutic strategies.

DNA methylation is one of the most well‐characterized epigenetic modifications that plays a crucial role in regulating gene expression and maintaining genomic stability [7, 8]. DNA methyltransferase 1 (DNMT1) is a key enzyme responsible for maintaining DNA methylation during cell division, ensuring the transmission of methylation patterns from parent to daughter cells [9]. Aberrant methylation, particularly hypermethylation of tumor suppressor genes and global hypomethylation, is commonly observed in malignant tumors and is closely associated with tumorigenesis, progression, and metastasis [10, 11, 12]. Understanding the molecular mechanisms by which DNMT1 contributes to oncogenesis may open new avenues for therapeutic intervention in PDAC.

Chromobox protein homolog 7 (CBX7), a component of the Polycomb repressive complex 1 (PRC1), plays a crucial role in chromatin remodeling and gene expression via epigenetic regulation [13, 14]. CBX7 regulates cellular senescence, proliferation, and differentiation by repressing key target genes [15, 16]. Its role in cancer is context‐dependent: it acts a tumor suppressor in cancers like colon and thyroid, where its downregulation correlates with poor prognosis and aggressive tumor behavior [17, 18]. Conversely, in malignancies such as lymphomagenesis, CBX7 exhibits oncogenic properties by repressing tumor suppressor genes [19]. This dual role suggests that its function is influenced by the cellular microenvironment and genetic context. Given the high mortality and limited therapeutic options for pancreatic cancer, understanding CBX7's role in its pathogenesis may reveal novel therapeutic targets.

In this study, we identified that DNMT1‐mediated hypermethylation of the CBX7 promoter results in the downregulation of CBX7 expression in PDAC, thereby suppressing PDAC tumorigenesis and metastasis through the inhibition of ERK signaling.

## 2 Materials and Methods

### 2.1 Patients and Specimens

Primary tumor specimens were collected from 24 patients diagnosed with pancreatic ductal adenocarcinoma who underwent complete surgical resection at Beijing Hospital between 2021 and 2023. Follow‐up data were obtained by reviewing the patients' medical records. Clinical characteristics and pathologic data associated with the patients are shown in Table S1. None of the patients received neoadjuvant chemotherapy or radiotherapy. This study was approved by the Ethics Committee of Beijing Hospital (2023BJYYEC‐240‐02), and all patients provided written informed consent in accordance with the Declaration of Helsinki.

### 2.2 Cell Culture

The human pancreatic ductal epithelial cell line HPDE and human pancreatic cancer cell lines PANC‐1, ASPC‐1, BxPC‐3, Capan‐1, MIAPaCa‐2, and CFPAC‐1 were obtained from the American Type Culture Collection. The culture of HPDE cells was carried out in DMEM containing 10% fetal bovine serum and 1% penicillin/streptomycin and maintained in a humidified incubator with 5% CO_2_ at 37°C.

### 2.3 Immunohistochemical Assay (IHC Assay)

Immunohistochemistry was performed on formalin‐fixed, paraffin‐embedded tissue sections from PDAC. Tissue sections were deparaffinized in xylene, rehydrated through a graded ethanol series, and subjected to antigen retrieval by autoclaving in 0.01 M citrate buffer (pH 6.0) for 20 min. Endogenous peroxidase activity was blocked with 0.3% hydrogen peroxide, and nonspecific binding was minimized by incubation with normal goat serum. The tissue sections were then incubated with primary antibodies at 4°C overnight. After incubating with the secondary antibody for 60 min, the specimens were processed with H_2_O_2_‐diaminobenzidine until the stain reached the required intensity. The antibodies used for the IHC assays are listed in Table S2.

The sections were counterstained with hematoxylin, dehydrated, and mounted. The intensity and extent of DNMT1 and CBX7 expression were graded as follows: negative (score 0), weak (score 1), moderate (score 2), and strong (score 3). Percentage coverage was scored as: 1 for 1%–25%, 2 for 26%–50%, 3 for 51%–75%, and 4 for 76%–100%. The intensity and percentage scores for each tumor sample were multiplied to obtain a final score ranging from 0 to 12. For statistical analysis, scores of 0 to 7 were considered low expression, and scores of 8 to 12 were considered high expression.

### 2.4 Western Blot

Protein expression levels were assessed by Western blot. Cells were lysed in RIPA buffer supplemented with protease and phosphatase inhibitors (Beyotime Biotechnology, China) on ice for 30 min, followed by centrifugation at 12000 × g for 15 min at 4°C to collect the supernatant. Protein concentrations were quantified using the BCA Protein Assay Kit (Thermo Fisher Scientific, USA). Equal amounts of protein were separated by 4%–12% sodium dodecyl sulfate‐polyacrylamide gel electrophoresis (SDS‐PAGE) and transferred onto PVDF membranes (Millipore, USA). Membranes were blocked with 5% non‐fat milk in TBS‐T (20 mM Tris–HCl, 150 mM NaCl, and 0.1% Tween‐20) for 1 h at room temperature, then incubated overnight at 4°C with primary antibodies targeting. Species‐matched secondary antibodies were subsequently applied to the membranes at room temperature. Protein bands were visualized using enhanced chemiluminescence (ECL) reagents (Lablead, China) and imaged with a chemiluminescence detection system. The antibodies used for the western blotting assays are listed in Table S2.

### 2.5 Real‐Time Polymerase Chain Reaction Analysis

Total RNA was extracted from cell samples using the RNAiso Plus (TaKaRa, Japan) according to the manufacturer's instructions. The concentration and purity of RNA were determined using a NanoDrop spectrophotometer (Thermo Fisher Scientific, USA). The reverse transcription was performed with the PrimeScript RT reagent Kit (TaKaRa, Japan). Quantitative real‐time PCR (qRT‐PCR) was conducted using SYBR Premix Ex Taq (TaKaRa, Japan) on a 7500 Real‐time PCR system (Applied Biosystems). Primer sequences were designed and validated to ensure specificity (listed in Table S3). The reaction conditions were as follows: initial denaturation at 95°C for 10 min, followed by 40 cycles of denaturation at 95°C for 15 s and annealing/extension at 60°C for 1 min. Relative mRNA expression levels were calculated using the 2^−ΔΔCt^ method and normalized to GAPDH as an internal control. All experiments were performed in triplicate to ensure reproducibility.

### 2.6 Cell Counting Kit‐8 (CCK‐8) Assay

Ten thousand single cells were seeded into each well of a 96‐well plate. After incubation for 24, 48, and 72 h, 100 μL of medium containing 10% CCK‐8 reagent (Beyotime Biotechnology, China) was added to each well. Six replicates were included for each group. Following a 30‐min incubation at 37°C, absorbance was measured at 450 nm using a Thermo Scientific Multiskan FC.

### 2.7 Wound Healing Assay

For detecting cell migration ability, cells were incubated on 12‐well plates. When the cell density reached 80% confluence, three scratches were made across the bottom of the plates in each well with a 200 μL pipette tip. The cells were then cultured in serum‐free medium. After 24 h, the scratch areas were photographed.

### 2.8 Transwell Assay

To assess cell invasion, PDAC cells in serum‐free medium were seeded into the upper chamber of Transwell inserts (8 μm pore size, Corning). The inserts were pre‐coated with Matrigel (Matrigel: Medium = 1:8). The lower chamber contained medium with 10% FBS as a chemoattractant. After incubation at 37°C for 48 h, cells on the upper membrane surface were removed. Cells on the lower surface were fixed with 4% paraformaldehyde and stained with 0.1% crystal violet. Invaded cells were counted in five random fields under a light microscope. Each experiment was performed in triplicate.

### 2.9 Dual‐Luciferase Reporter Assays

The CBX7 promoter constructs were inserted into the pGL3‐Basic vector utilizing the Rapid DNA Ligation Kit (Thermo Scientific). The vector pcDNA3.1‐DNMT1 overexpressed DNMT1, and the corresponding empty vector pcDNA3.1‐NC (vector2) was constructed by Genomeditech. All constructs were validated through sequencing. For the luciferase reporter assay, cells were seeded in 12‐well plates and incubated for 24 h prior to transfection with luciferase reporter constructs and the Renilla luciferase plasmid. After 48 h, cells were harvested, and luciferase activity was quantified using the Dual‐Luciferase Reporter Assay System (Promega), following the manufacturer's protocol. In brief, cells were collected and washed with phosphate‐buffered saline (PBS). Subsequently, 500 μL of Passive Lysis Buffer (Promega) was added to each well, and the plates were gently rocked at room temperature for 15 min. A 10 μL aliquot of the lysate was then transferred to a black 96‐well plate (Thermo) for analysis. Sequential assays were conducted to measure Firefly and Renilla luciferase activities in the cell lysate of each well. Transcriptional activity was quantified by calculating the ratio of Firefly luciferase activity (reporter) to Renilla luciferase activity (control).

### 2.10 Methylation‐Specific PCR (MS‐PCR)

Genome DNA was extracted using the Universal Genomic DNA Extraction Kit (Tiangen Biotech, China). Subsequently, DNA samples were assayed for methylation levels using the DNA Bisulfite Conversion Kit (Tiangen Biotech, China) and the Methylation‐Specific PCR Kit (Tiangen Biotech, China), following the instructions provided by the manufacturer. The methylation and non‐methylation primers for CBX7 are listed in Table S4.

### 2.11 Chromatin Immunoprecipitation (ChIP)

Chromatin immunoprecipitation (ChIP) was performed using the ChIP Assay Kit (Magnetic Beads) (Cell Signaling Technology, Cat. No. 9005). Cells were fixed with 1% formaldehyde at RT for 10 min, followed by two washes with ice‐cold PBS containing 1 mM PMSF. The cells were then collected and resuspended in 0.2 mL of ice‐cold 1X Buffer A with 1 mM PMSF and DTT, followed by lysis on ice for 10 min to ensure thorough cell disruption. Micrococcal nuclease was added, mixed by inverting the tube several times, and incubated at 37°C for 20 min with frequent mixing to digest DNA into fragments approximately 150–900 bp in length. Samples were incubated overnight at 4°C with 2 μg of DNMT1‐antibody, IgG‐antibody (negative control), or 10 μL of H3‐antibody (positive control). Add 30 μL of Protein G Magnetic Beads to each IP reaction and incubate for 2 h at 4°C with rotation. Beads were washed sequentially with low‐salt wash buffer, with each wash performed for 5 min at 4°C. Pellet protein G magnetic beads by placing the tubes in a magnetic separation rack. Immune complexes were eluted using 50 μL of DNA Elution Buffer, and the DNA was recovered by centrifugation in a microcentrifuge at 18000 × g for 30 s. Samples were treated with 10 μL of 0.5 M EDTA, 20 μL of 1 M Tris (pH 6.5), and 1 μL of 20 mg/mL proteinase K to remove proteins. DNA was purified through phenol and phenol/chloroform extraction, and the percentage of chromatin‐bound DNA was quantified relative to input DNA.

### 2.12 Statistical Analyses

All data are presented as mean ± SD from at least three independent biological experiments.

Statistical analysis of pancreatic cancer tissue staining was conducted using SPSS version 20. The Student's *t*‐test was applied to evaluate the statistical significance between two groups, with a significance threshold set at *p* < 0.05. Additional statistical analyses were performed using GraphPad Prism software.

## 3 Results

### 3.1 DNMT1 Expression Is Upregulated in PDAC Tissues and Cells, and Is Associated With the Malignant Progression of PDAC Cells

**FIGURE 1:**
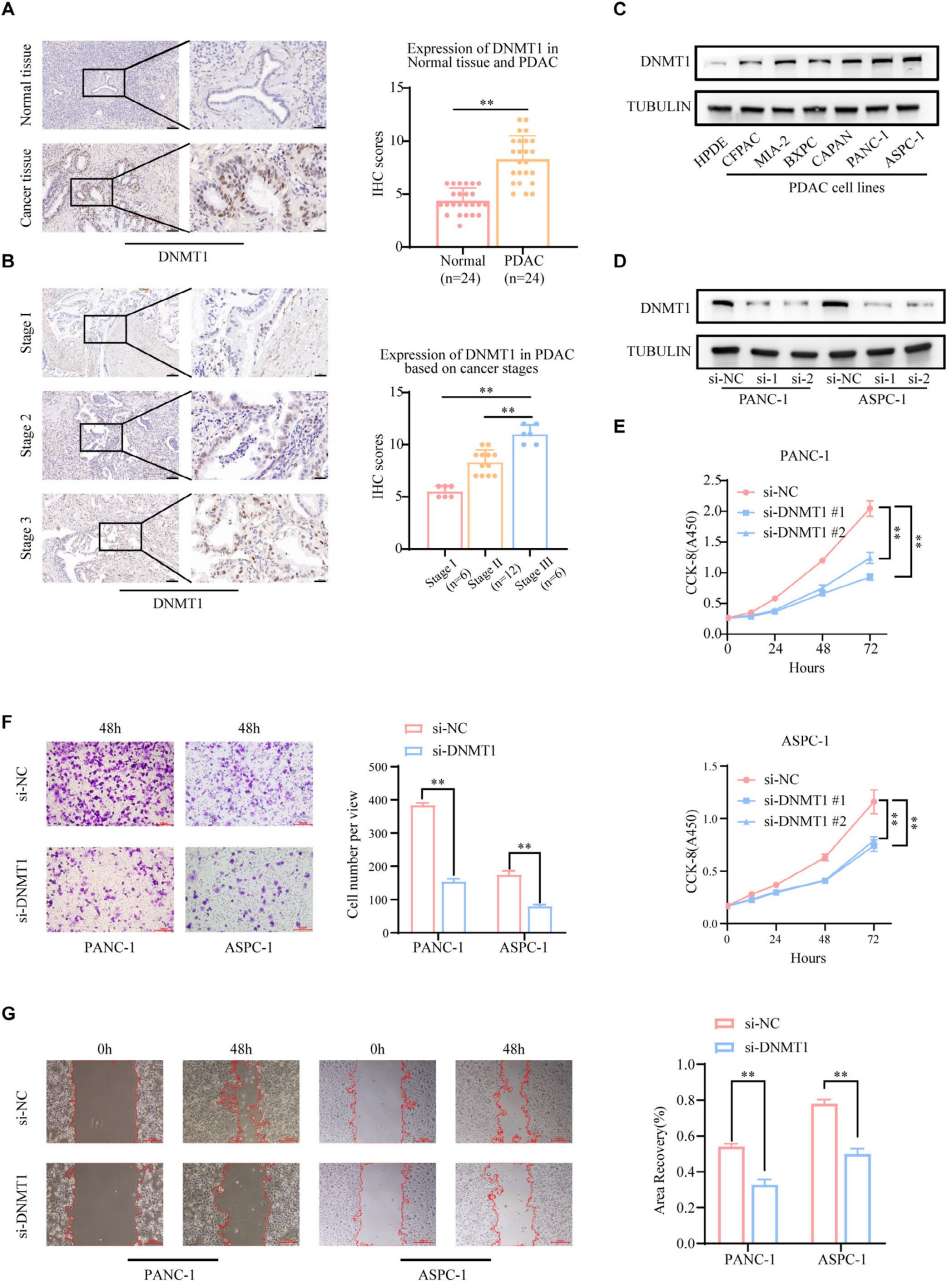
DNMT1 expression is upregulated in PDAC tissues and cells, and is associated with the malignant progression of PDAC cells. (A) IHC examined DNMT1 expression in PDAC tissues and adjacent normal pancreatic tissues. The scale bar indicated in panel (B) was 100 and 10 μm, respectively. (B) The IHC scoring was performed, and the correlation between IHC score of CBX7 and cancer stage was assessed. The scale bar indicated in panel (B) was 100 and 10 μm, respectively. (C) DNMT1 expression in the human pancreatic duct epithelial cell line (HPDE) and a panel of PDAC cell lines was measured by western blot analysis. (D) PANC‐1 and ASPC‐1 cells were transfected with si‐NC, siDNMT1‐1, or siDNMT1‐2 for 48 h. Then, cells were collected for western blot analysis. (E–G) PANC‐1 and ASPC‐1 cells were infected with indicated si‐NC, siDNMT1‐1, or siDNMT1‐2 for 48 h. Cells were harvested for CCK‐8 assay, transwell assay, and wound healing assay. Data presented as the mean ± SD of three independent experiments. ***p < 0.001.

Firstly, IHC analysis revealed that DNMT1 expression was significantly elevated in PDAC tissues compared to adjacent normal tissues (Figure 1A). Furthermore, a significant correlation was observed between DNMT1 expression and TNM stage (*p* < 0.05), with expression levels increasing progressively with higher TNM stages (Figure 1B), suggesting that DNMT1 expression may increase progressively as PDAC progresses, potentially contributing to tumor aggressiveness and severity.

Next, we investigated the expression of DNMT1 in 6 pancreatic cancer cell lines and 1 human pancreatic ductal epithelial cell line HPDE by qRT‐PCR and western blot. Furthermore, compared with HPDE, DNMT1 was up‐regulated in PDAC cells. PANC‐1 and ASPC‐1 were the most obvious cell lines (Figure 1C and Figure S1A).

Finally, to investigate the role of DNMT1 in PDAC, we knocked down its expression in PDAC cell lines (PANC‐1 and ASPC‐1) using siRNA. Compared to control cells, DNMT1 was obviously reduced in cells with DNMT1 silencing (Figure 1D). Besides, after down‐regulation of DNMT1 expression, cell proliferation, migration, and invasion abilities were all impaired (Figure 1E–G). The above data suggest that DNMT1 plays a critical role in PDAC progression.

### 3.2 Integrative Bioinformatics and Experimental Analysis Reveal CBX7 as a Key DNMT1‐Associated Gene in PDAC

**FIGURE 2:**
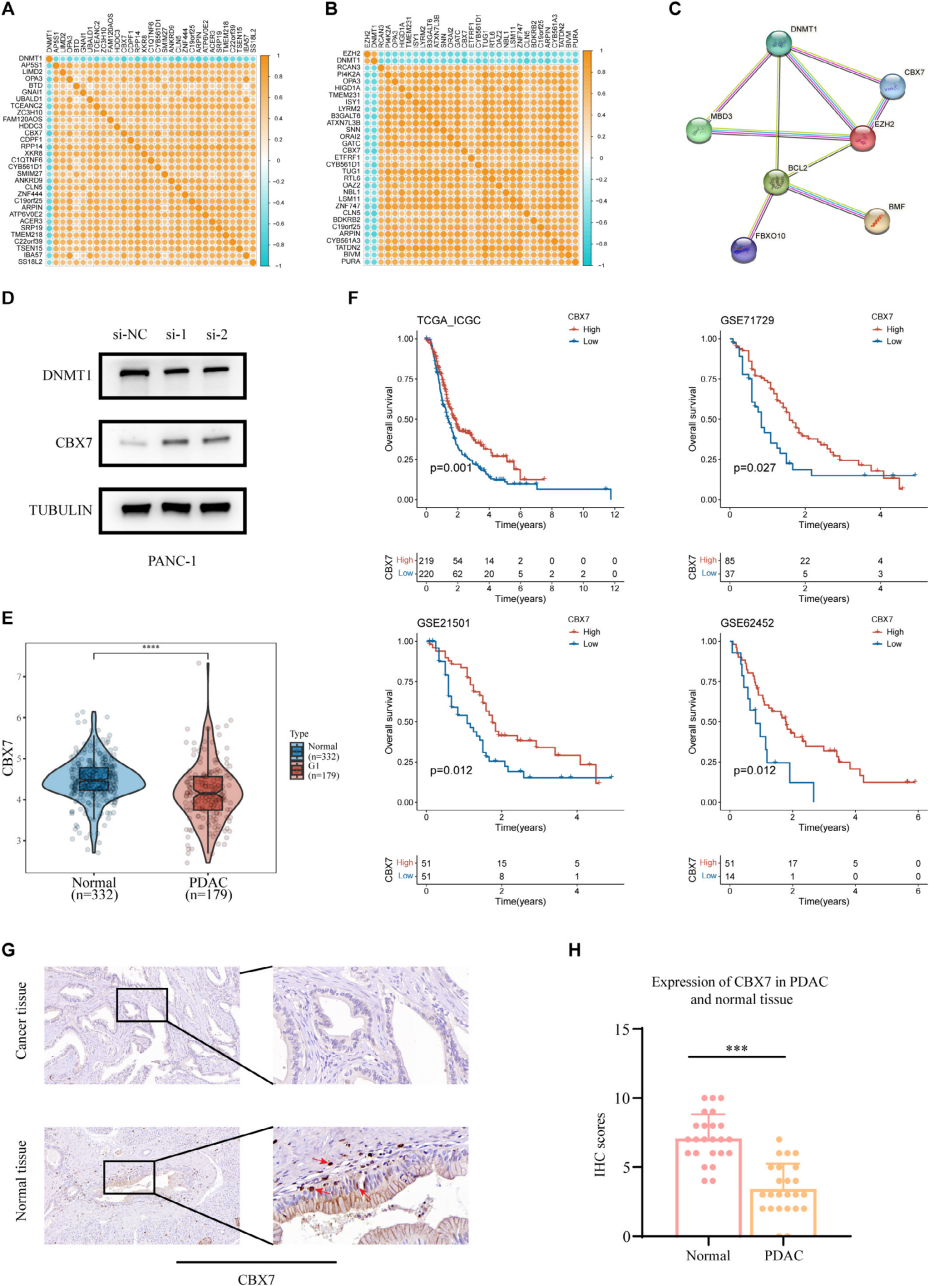
Integrative Bioinformatics and Experimental Analysis Reveal CBX7 as a Key DNMT1‐Associated Gene in PDAC. (A) A total of 5048 genes were identified as strongly associated with DNMT1 through a correlation analysis conducted on DNMT1 and all mRNAs in the TCGA‐ICGC dataset. (B) Correlation analysis of DNMT1‐HRGs and EZH2 identified 159 genes with a negative correlation, as calculated using the method described above. (C) Correlation analysis identified mRNAs highly correlated with DNMT1 and EZH2 expression (|correlation coefficient| > 0.6), followed by PPI network analysis using STRING (https://cn.string‐db.org/). (D) PANC‐1 cells were transfected with si‐NC, siDNMT1‐1, or siDNMT1‐2 for 48 h. Then, cells were collected for western blot analysis. (E) CBX7 mRNA expression between PDAC tissues and the normal tissues in TCGA and GTEx. (F) Kaplan‐Meier plot of overall survival of PDAC patients in TCGA_ICGC, GSE71729, GSE21501, and GSE62452. (G‐H) IHC examined CBX7 expression in PDAC tissues and adjacent normal pancreatic tissues. The scale bar indicated in panel (B) was 100 and 10 μm, respectively. ***p < 0.001.

In order to identify DNMT1 highly related genes (DNMT1‐HRGs), we used the TCGA‐ICGC data set to conduct correlation analysis on DNMT1 and all mRNAs, used the spearman correlation test to calculate the correlation coefficient, and selected genes with an absolute value of the correlation coefficient > 0.6 for subsequent research. As a result, 5048 genes were considered to be highly related to DNMT1(Figure 2A). Further correlation analysis was performed on DNMT1‐HRGs and EZH2, and the correlation coefficient calculation method was as described above (Figure 2B). Ultimately, 159 genes were selected. We included the above 159 genes, DNMT1, and EZH2 for Protein–Protein Interaction (PPI) analysis using STRING (https://cn.string‐db.org/), and adjusted the protein interaction correlation coefficient to 0.6 (Figure 2C) The results showed that CBX7 has a strong association with DNMT1.

To verify the results of bioinformatics analysis, we analyzed the effect of downregulated DNMT1 expression in PANC‐1 cells in vitro. We observed that DNMT1 expression was notably silenced whereas CBX7 expression was raised in PANC‐1 cells following treatment with si‐DNMT1(Figure 2D). Meanwhile, we transfected PANC‐1 cells with pCMV‐DNMT1 and control plasmids. The Western blot results showed a significant decrease in CBX7 expression following DNMT1 overexpression (Figure S1B). As a result, CBX7 was selected for further investigation. To determine whether CBX7 expression correlates with the prognosis of PDAC, we conducted a differential expression analysis utilizing data from The Cancer Genome Atlas (TCGA), comparing CBX7 expression levels between adjacent normal tissues and PDAC tissues. The analysis indicated a significant upregulation of DNMT1 expression in PDAC tissues relative to adjacent normal tissues (Figure 2E). Additionally, we performed survival analysis of CBX7 across multiple PDAC datasets, generating corresponding survival curves (Figure 2F). The findings demonstrated that patients with high CBX7 expression exhibited significantly improved overall survival compared to those with low CBX7 expression. Finally, we wanted to evaluate the clinical significance of our findings in PDAC. We assessed CBX7 expression in PDAC tissues, and IHC analysis revealed that CBX7 was significantly downregulated in PDAC tissues compared to adjacent normal tissues (Figure 2G,H).

### 3.3 CBX7 Overexpression Inhibits PDAC Cell Proliferation, Migration, and Invasion

**FIGURE 3:**
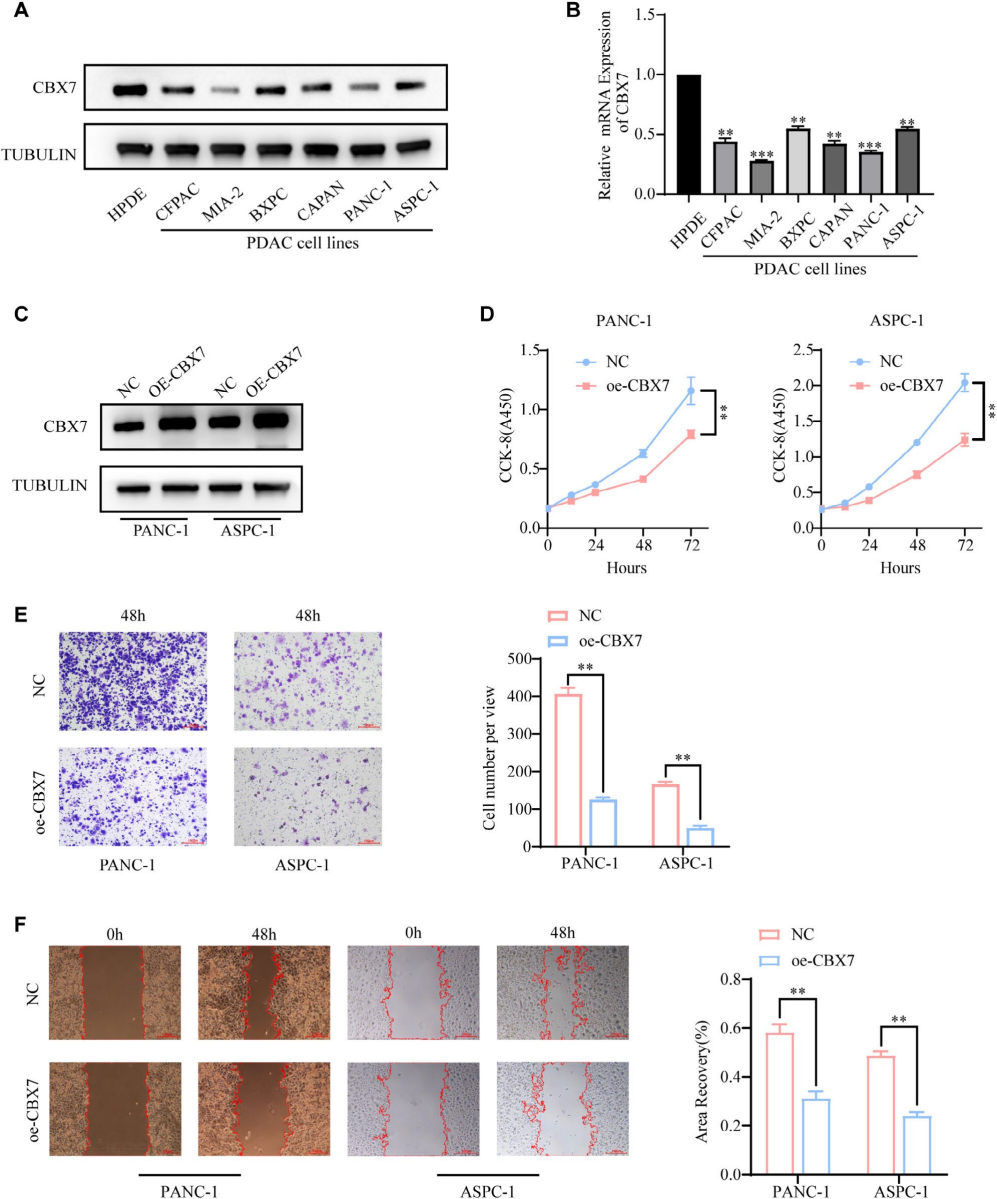
CBX7 overexpression inhibits PDAC cell proliferation, migration, and invasion, while promoting apoptosis. (A, B) CBX7 expression in the HPDE cell line and a panel of PDAC cell lines was measured by (A) western blot analysis and (B) qRT‐PCR analysis. (C) PANC‐1 and ASPC‐1 cells were transfected with the pCDH‐CBX7 expression vector for 48 h, and CBX7 expression was measured by western blot. (D–F) PANC‐1 and ASPC‐1 cells were transfected with the pCDH‐CBX7 expression vector for 48 h. Cells were harvested for CCK‐8 assay, transwell assay, and wound healing assay. Data presented as the mean ± SD of three independent experiments. **p < 0.01, ***p < 0.001.

We investigated the expression of CBX7 in 6 pancreatic cancer cell lines and 1 human pancreatic ductal epithelial cell line HPDE by qRT‐PCR and western blot. CBX7 was down‐regulated in PDAC cells (Figure 3A,B).

For assessment of the influence of CBX7 on PDAC tumor growth, PANC‐1 and ASPC‐1 cells were pre‐transfected with pCDH‐CBX7 and control plasmid. First, a CCK‐8 assay showed that overexpression of CBX7 (OE‐CBX7) decreased the proliferation of PDAC cells (Figure 3C,D). Meanwhile, we also found that CBX7 overexpression inhibited the migration and invasion ability of PANC‐1 and ASPC‐1 cells in comparison to the control group (Figure 3E,F).

### 3.4 DNMT1 Suppresses CBX7 Expression by Promoting Methylation of the CBX7 Promoter CpG Island Region

**FIGURE 4:**
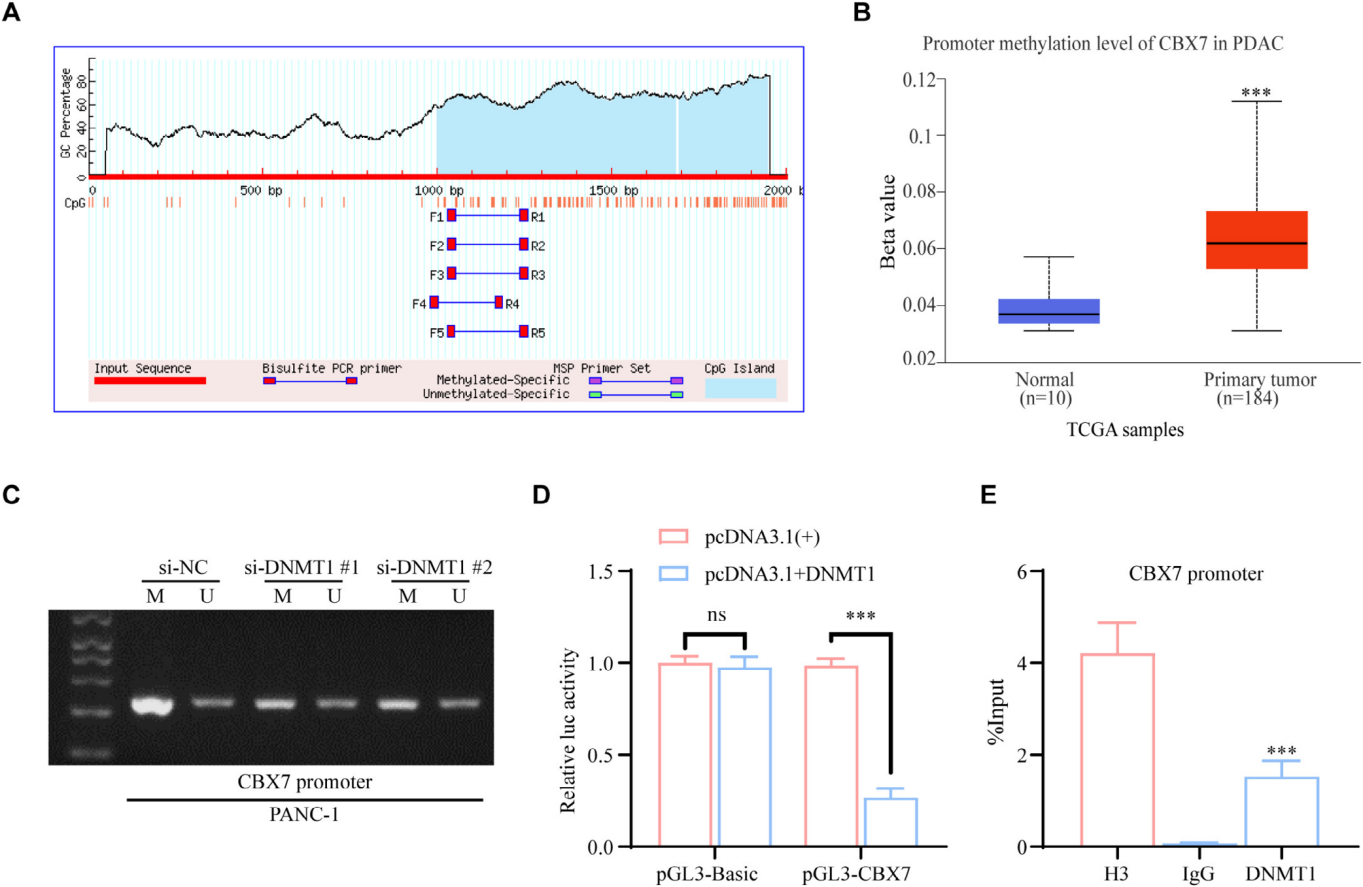
DNMT1 suppresses CBX7 expression by promoting methylation of the CBX7 promoter CpG island region. (A) Schematic representation of CBX7 promoter CpG islands and bisulfite sequencing region in the CBX7 promoter. (B) The UALCAN (http://ualcan.path.uab.edu/index.html) website was used to analyze the correlation between promoter methylation and expression of CBX7 in PDAC. (C)Methylation status of the CBX7 promoter was evaluated in DNMT1 knockdown cells using methylated (M) and unmethylated (U) primers. Methylation was determined by PCR agarose gel electrophoresis. (D) Luciferase reporter assays detected the CBX7 promoter region activity after ectopic expression of DNMT1 in PANC‐1 cells. (E) ChIP assays on the CBX7 promoter were done using anti‐DNMT1 antibody in PANC‐1 cells and were analyzed by qRT‐PCR. DNA fragments obtained without antibody served as input controls. Histone H3 antibodies were used as the positive control in ChIP assays, with IgG as the negative control. ***p < 0.001.

Our findings have established that DNMT1 represses the expression of CBX7 in pancreatic cancer, and that CBX7 contributes to the inhibition of pancreatic cancer progression. Consequently, we intend to conduct further research to elucidate the mechanisms by which DNMT1 regulates CBX7 expression within this context. Using the MethPrimer 2.0 online platform (http://www.urogene.org/methprimer2/), we discovered a CpG island in the promoter region of CBX7(Figure 4A). The TCGA dataset also revealed that promoter methylation was increased in primary PDAC tissues, compared to normal tissue (Figure 4B). DNMT1 is widely recognized for its ability to inhibit the transcription of target genes by facilitating DNA methylation on the CpG island regions of promoters. Consequently, we assumed that DNMT1 downregulates CBX7 transcription by inducing DNA methylation in the promoter's CpG island region.

To test our hypothesis, we first conducted DNA methylation PCR assays to assess changes in DNA methylation following DNMT1 knockdown. The results demonstrated a significant reduction in DNA methylation levels in the DNMT1 knockdown group (si‐DNMT1 #1 and si‐DNMT1 #2) compared with the control group (si‐NC) (Figure 4C). Luciferase reporter assays were then used to assess DNMT1's impact on CBX7 transcription, revealing that DNMT1 inhibits CBX7 promoter activity (Figure 4D). ChIP revealed that DNMT1 protein directly binds to the CBX7 promoter in PANC‐1 cells. (Figure 4E). These findings indicate that DNMT1 downregulates CBX7 expression via promoter DNA methylation.

### 3.5 DNMT1 Promotes the Viability of PDAC Cells via the CBX7/ERK Signaling Pathway

**FIGURE 5:**
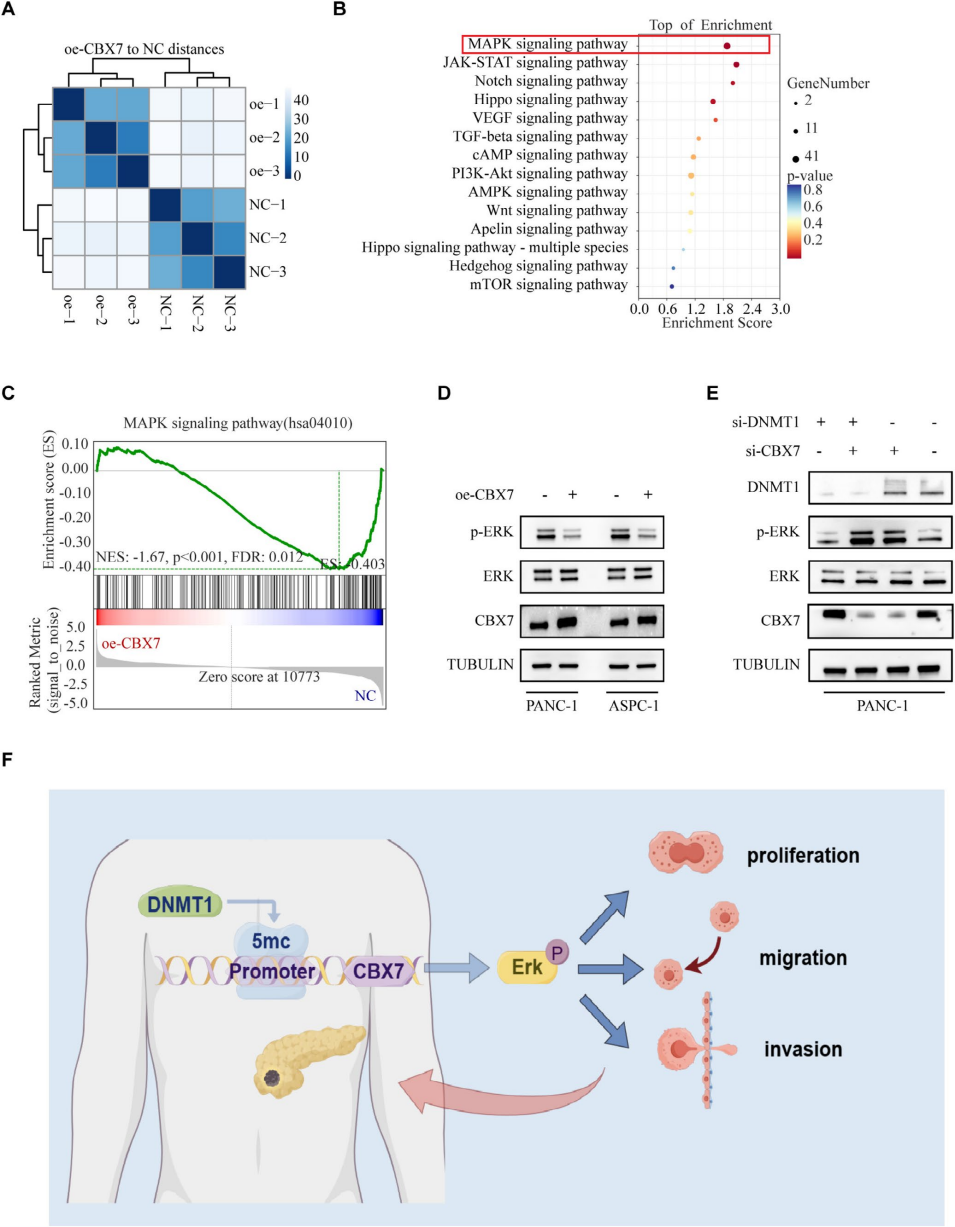
DNMT1 promotes the viability of PDAC cells via the CBX7/ERK signaling pathway. (A) This heatmap shows the clustering of transcriptomes from two groups: CBX7‐overexpressing (oe‐CBX7) PANC‐1 and control cells (NC), each with three biological replicates (oe‐CBX7: Oe‐1, oe‐2, oe‐3; NC: NC‐1, NC‐2, NC‐3). (B) KEGG enrichment analysis of RNA‐seq data between the oe‐CBX7 and NC groups. *p*‐values as indicated. (C) GSEA analysis of RNA‐seq data of CBX7 from the PANC‐1 cells. *p*‐values as indicated. (D) PANC‐1 and ASPC‐1 cells were transfected with pCDH‐CBX7 expression vector for 48 h. Cells were harvested for western blot analysis. (E) PANC‐1 cells were transfected with si‐DNMT1 + si‐NC, si‐DNMT1 + si‐CBX7, si‐NC + si‐CBX7, or si‐NC + si‐NC for 48 h. Then, cells were harvested for western blot analysis. (F) A working model of the DNMT1/CBX7/ERK axis in PDAC progression by Figdraw.

To further analyze how overexpressing CBX7 influences transcriptional responses, transcriptional profiling by RNA‐seq was conducted with CBX7‐overexpressing (oe‐CBX7) PANC‐1 and control cells (NC) (Figure 5A). KEGG enrichment pathway analysis indicated that there was a close relationship between CBX7 and the MAPK signaling pathway (Figure 5B). Moreover, GSEA analysis of the RNA‐seq data also suggested that overexpression of CBX7 was negatively correlated with activation of the MAPK signaling pathway in the PDAC cell line (PANC‐1) (Figure 5C). Consistently, we showed that overexpression of CBX7 in PDAC cells (PANC‐1 and ASPC‐1 cells) decreased the phosphorylation of ERK (Figure 5D). To elucidate the function of CBX7 in the DNMT1‐promoting PDAC progression, we performed rescue experiments in si‐DNMT1 cells by knockdown of CBX7 expression by transfection of siRNA (Figure 5E). We found that ERK phosphorylation levels significantly decreased following DNMT1 knockdown. However, upon further knockdown of CBX7 in the si‐DNMT1 background, ERK phosphorylation levels were restored to previous levels. Our findings, along with the rescue experiments, suggest that reducing CBX7 expression is necessary for DNMT1 to enhance PDAC progression through ERK signaling (Figure 5F).

## 4 Discussion

PDAC is characterized by highly aggressive biological behavior and poor prognosis. Therefore, elucidating the molecular mechanisms underlying its progression is essential for advancing diagnostic and therapeutic strategies [5, 20]. Our study reveals that DNMT1 contributes to PDAC progression by suppression of CBX7 expression. Additionally, we identified a significant association between DNMT1 and CBX7 expression in PDAC patients. Mechanistically, DNMT1‐mediated suppression of CBX7 activates the extracellular signal‐regulated kinase (ERK) pathway through increased ERK phosphorylation, thereby facilitating tumor growth and progression. This regulatory axis suggests that DNMT1‐mediated epigenetic modifications play a critical role in driving PDAC malignancy by modulating key oncogenic signaling pathways.

Recent studies increasingly demonstrate that DNA hypermethylation significantly impacts cancer progression across various malignancies [21]. This hypermethylation results in the silencing of critical tumor suppressor genes, thereby promoting tumor development and progression. The overexpression of DNMT1 has been implicated in multiple cancers, including pancreatic cancer, where it shapes an epigenetic landscape that favors tumor growth and metastasis [22, 23]. Research shows that targeting DNMT1 can reverse the hypermethylation of these promoter regions, potentially restoring the expression of tumor suppressor genes and inhibiting cancer progression. For instance, Meng J et al. found that DNMT1 overexpression induced by ITGA2 silencing significantly upregulated the methylation level of the STING gene promoter, and therefore suppress the anti‐tumor immune response [24]. Furthermore, the interaction between DNMT1 and other signaling pathways, such as the PI3K‐AKT pathway, highlights its complex role in cancer biology. Lin et al. demonstrated that the DNMT1 inhibitor 5‐azacytidine (5‐Aza), when combined with gefitinib or osimertinib, effectively restored drug sensitivity in non‐small cell lung cancer (NSCLC) patients [25]. These findings underscore the complexity of DNMT1's involvement in oncogenic processes and support the potential of combination therapies for improved treatment outcomes. Our study provides evidence that DNMT1 significantly contributes to the progression of pancreatic ductal adenocarcinoma (PDAC). Through its role in promoting epigenetic silencing of tumor suppressor genes, DNMT1 facilitates tumor growth and invasion, underscoring its potential as a critical driver of PDAC malignancy.

The silencing of CBX7 in PDAC suggests that CBX7 could function as a tumor suppressor, and its reduced expression likely aids in the development and progression of PDAC. Karamitopoulou et al. found that loss of CBX7 expression was associated with an increasing malignancy grade in pancreatic adenocarcinoma, whereas the maintenance of CBX7 expression correlated with longer survival [26]. There are similar studies in the literature with our results. Studies have shown that CBX7 negatively modulates PTEN/Akt signaling during pancreatic tumorigenesis by upregulating PTEN transcription, suggesting that the PTEN/Akt pathway mediates the tumor‐suppressive activity of CBX7 [27]. In our study, RNA‐seq analysis revealed a close association between CBX7 and the MAPK signaling pathway in PDAC. Western blot experiments further confirmed that CBX7 inhibits PDAC progression by suppressing ERK phosphorylation. Notably, Zhengnan H et al. found that in bladder cancer, CBX7 functions as a tumor suppressor by downregulating AKR1B10, which leads to the inactivation of ERK signaling[28]. Collectively, the results provide strong evidence that CBX7 acts as a critical tumor suppressor in PDAC.

In summary, our study reveals that DNMT1‐mediated methylation of the CBX7 promoter region leads to the downregulation of CBX7 expression in PDAC, which is significantly associated with tumor clinical staging. Notably, we demonstrate for the first time that CBX7 exerts its tumor‐suppressive function in PDAC by inhibiting ERK phosphorylation, thereby suppressing cell proliferation, migration, and invasion. This newly identified DNMT1/CBX7/ERK axis holds substantial promise as a potential target for the development of novel therapeutic strategies against PDAC.

## Author Contributions

H.S., X.L., and J.S. conceived and designed the experiments. H.S., Y.G., and W.T. contributed reagents/materials/analysis tools and reviewed drafts of the paper. S.G. and Y.Z. collected data and clinical samples. X.L. and C.X. analyzed the data and prepared figures and/or tables. C.X., J.X., Y.L., and J.S. contributed, authored, or reviewed drafts of the paper and approved the final draft.

## Ethics Statement

This study adheres to ethical standards and was ensured by conducting the study in compliance with the Declaration of Helsinki (revised in 2013), and ethical approval was secured from the Hospital Ethics Committee of the Beijing Hospital (2023BJYYEC‐240‐02). Each participant has signed a detailed written consent form.

## Consent

The authors have nothing to report.

## Conflicts of Interest

The authors declare no conflicts of interest.

## Supporting information


Figure S1.

Table S1.

Table S2.

Table S3.

Table S4.

## Data Availability

All data used in this study are available from the authors on reasonable request.

## References

[1] F. Bray , M. Laversanne , H. Sung , et al., “Global Cancer Statistics 2022: GLOBOCAN Estimates of Incidence and Mortality Worldwide for 36 Cancers in 185 Countries,” CA: A Cancer Journal for Clinicians 74, no. 3 (2024): 229–263. 38572751 10.3322/caac.21834

[2] L. Rahib , B. D. Smith , R. Aizenberg , A. B. Rosenzweig , J. M. Fleshman , and L. M. Matrisian , “Projecting Cancer Incidence and Deaths to 2030: The Unexpected Burden of Thyroid, Liver, and Pancreas Cancers in the United States,” Cancer Research 74, no. 11 (2014): 2913–2921. 24840647 10.1158/0008-5472.CAN-14-0155

[3] S. Rebecca L , G. Angela N , and J. Ahmedin , “Cancer Statistics, 2024,” CA: A Cancer Journal for Clinicians 74, no. 1 (2024): 12–49. 38230766 10.3322/caac.21820

[4] C. J. Halbrook , C. A. Lyssiotis , M. P. di Magliano , and A. Maitra , “Pancreatic Cancer: Advances and Challenges,” Cell 186, no. 8 (2023): 1729–1754. 37059070 10.1016/j.cell.2023.02.014 PMC10182830

[5] Z. I. Hu and E. M. O'Reilly , “Therapeutic Developments in Pancreatic Cancer,” Nature Reviews. Gastroenterology & Hepatology 21, no. 1 (2023): 7–24. 37798442 10.1038/s41575-023-00840-w

[6] M. H. Sherman and G. L. Beatty , “Tumor Microenvironment in Pancreatic Cancer Pathogenesis and Therapeutic Resistance,” Annual Review of Pathology: Mechanisms of Disease 18 (2022): 123–148. 10.1146/annurev-pathmechdis-031621-024600 PMC9877114 36130070

[7] F. Liu , Y. Wang , H. Gu , and X. Wang , “Technologies and Applications of Single‐Cell DNA Methylation Sequencing,” Theranostics 13, no. 8 (2023): 2439–2454. 37215576 10.7150/thno.82582 PMC10196823

[8] M. V. C. Greenberg and D. Bourc'his , “The Diverse Roles of DNA Methylation in Mammalian Development and Disease,” Nature Reviews. Molecular Cell Biology 20, no. 10 (2019): 590–607. 31399642 10.1038/s41580-019-0159-6

[9] Z. D. Smith and A. Meissner , “DNA Methylation: Roles in Mammalian Development,” Nature Reviews. Genetics 14, no. 3 (2013): 204–220. 10.1038/nrg3354 23400093

[10] M. Ehrlich , “DNA Methylation in Cancer: Too Much, but Also Too Little,” Oncogene 21, no. 35 (2002): 5400–5413. 12154403 10.1038/sj.onc.1205651

[11] Y. Yin , E. Morgunova , A. Jolma , et al., “Impact of Cytosine Methylation on DNA Binding Specificities of Human Transcription Factors,” Science (New York, N.Y.) 356, no. 6337 (2017): 502–517. 10.1126/science.aaj2239 PMC8009048 28473536

[12] D. Jjingo , A. B. Conley , S. V. Yi , V. V. Lunyak , and I. K. Jordan , “On the Presence and Role of Human Gene‐Body DNA Methylation,” Oncotarget 3, no. 4 (2012): 462–474. 22577155 10.18632/oncotarget.497 PMC3380580

[13] R. G. Ma , Y. Zhang , T. T. Sun , and B. Cheng , “Epigenetic Regulation by Polycomb Group Complexes: Focus on Roles of CBX Proteins,” Journal of Zhejiang University. Science. B 15, no. 5 (2014): 412–428. 24793759 10.1631/jzus.B1400077 PMC4076598

[14] M. A. Iqbal , S. Siddiqui , A. Ur Rehman , et al., “Multiomics Integrative Analysis Reveals Antagonistic Roles of CBX2 and CBX7 in Metabolic Reprogramming of Breast Cancer,” Molecular Oncology 15, no. 5 (2021): 1450–1465. 33400401 10.1002/1878-0261.12894 PMC8096797

[15] J. Li , A. B. Alvero , S. Nuti , et al., “CBX7 Binds the E‐Box to Inhibit TWIST‐1 Function and Inhibit Tumorigenicity and Metastatic Potential,” Oncogene 39, no. 20 (2020): 3965–3979. 32205869 10.1038/s41388-020-1269-5 PMC8343988

[16] K. L. Yap , S. Li , A. M. Muñoz‐Cabello , et al., “Molecular Interplay of the Noncoding RNA ANRIL and Methylated Histone H3 Lysine 27 by Polycomb CBX7 in Transcriptional Silencing of INK4a,” Molecular Cell 38, no. 5 (2010): 662–674. 20541999 10.1016/j.molcel.2010.03.021 PMC2886305

[17] P. Pallante , A. Federico , M. T. Berlingieri , et al., “Loss of the CBX7 Gene Expression Correlates With a Highly Malignant Phenotype in Thyroid Cancer,” Cancer Research 68, no. 16 (2008): 6770–6778. 18701502 10.1158/0008-5472.CAN-08-0695

[18] P. Pallante , L. Terracciano , V. Carafa , et al., “The Loss of the CBX7 Gene Expression Represents an Adverse Prognostic Marker for Survival of Colon Carcinoma Patients,” European Journal of Cancer 46, no. 12 (2010): 2304–2313. 20542683 10.1016/j.ejca.2010.05.011

[19] C. L. Scott , J. Gil , E. Hernando , et al., “Role of the chromobox protein CBX7 in lymphomagenesis,” Proc Natl Acad Sci USA 104, no. 13 (2007): 5389–5394. 17374722 10.1073/pnas.0608721104 PMC1828941

[20] A. Mehdi and S. A. Rabbani , “Role of Methylation in pro‐ and Anti‐Cancer Immunity,” Cancers 13, no. 3 (2021): 545–570. 33535484 10.3390/cancers13030545 PMC7867049

[21] H. Li , J. W. Liu , L. P. Sun , and Y. Yuan , “A Meta‐Analysis of the Association Between Polymorphisms and Cancer Risk,” BioMed Research International 2017 (2017): 3971259. 28473984 10.1155/2017/3971259 PMC5394348

[22] K. K. Wong , “DNMT1 as a Therapeutic Target in Pancreatic Cancer: Mechanisms and Clinical Implications,” Cellular Oncology (Dordrecht, Netherlands) 43, no. 5 (2020): 779–792. 32504382 10.1007/s13402-020-00526-4 PMC12990712

[23] J. Meng , H. Cai , Y. Sun , S. Wen , H. Wu , and D. Ren , “ITGA2 Induces STING Expression in Pancreatic Cancer by Inducing DNMT1 Degradation,” Cellular Oncology 45, no. 6 (2022): 1421–1434. 36331797 10.1007/s13402-022-00731-3 PMC12978104

[24] S. Lin , H. Ruan , L. Qin , et al., “Acquired Resistance to EGFR‐TKIs in NSCLC Mediates Epigenetic Downregulation of MUC17 by Facilitating NF‐κB Activity via UHRF1/DNMT1 Complex,” International Journal of Biological Sciences 19, no. 3 (2023): 832–851. 36778111 10.7150/ijbs.75963 PMC9910003

[25] E. Karamitopoulou , P. Pallante , I. Zlobec , et al., “Loss of the CBX7 Protein Expression Correlates With a More Aggressive Phenotype in Pancreatic Cancer,” European Journal of Cancer 46, no. 8 (2010): 1438–1444. 20185297 10.1016/j.ejca.2010.01.033

[26] S. Ni , H. Wang , X. Zhu , et al., “CBX7 Suppresses Cell Proliferation, Migration, and Invasion Through the Inhibition of PTEN/Akt Signaling in Pancreatic Cancer,” Oncotarget 8, no. 5 (2017): 8010–8021. 28030829 10.18632/oncotarget.14037 PMC5352378

[27] H. Z , Z. Huang , Y. Yan , et al., “CBX7 Suppresses Urinary Bladder Cancer Progression via Modulating AKR1B10‐ERK Signaling,” Cell Death & Disease 12, no. 6 (2021): 537, 10.1038/s41419-021-03819-0. 34035231 PMC8149849

