# Supplementary figures and images for: DNMT1‐Induced Downregulation of CBX7 Inhibits ERK Phosphorylation and Promotes Pancreatic Ductal Adenocarcinoma Progression
Source: FASEB J. 2025 May 19;39(10):e70571. doi: 10.1096/fj.202402903R (PMC12087528; doi:10.1096/fj.202402903R)

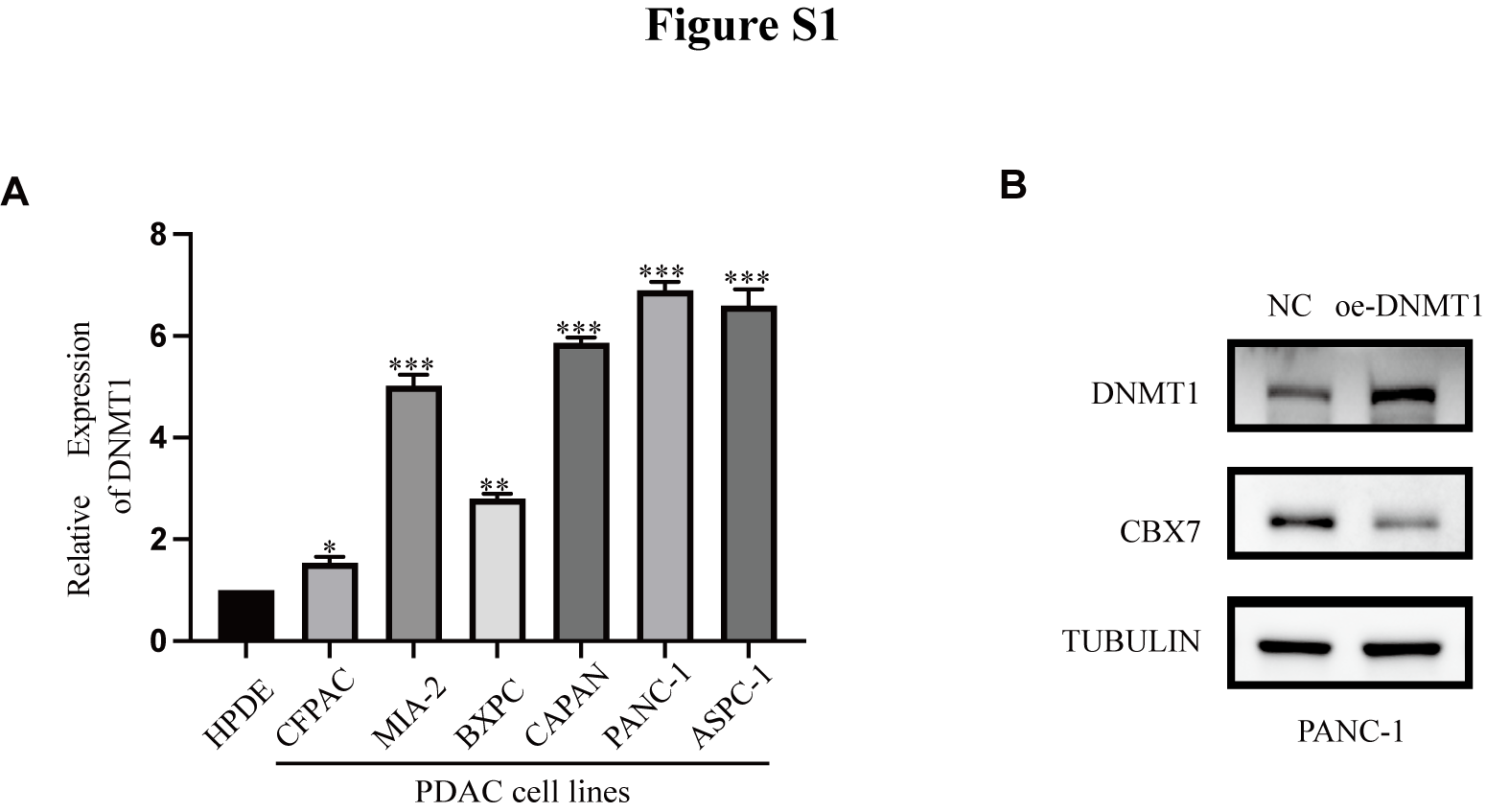

Supplement: Supplementary file 1 — Figure S1. [file FSB2-39-e70571-s004.tif]
