# Supplementary material for: DNMT1‐Induced Downregulation of CBX7 Inhibits ERK Phosphorylation and Promotes Pancreatic Ductal Adenocarcinoma Progression
Source: FASEB J. 2025 May 19;39(10):e70571. doi: 10.1096/fj.202402903R (PMC12087528; doi:10.1096/fj.202402903R)
Supplement: Supplementary file 2 — Table S1. [file FSB2-39-e70571-s001.docx]

**Table S1.** **The association between DNMT1 protein levels and clinicopathological features of PDAC patients (n=24)**

|  | | **Expression of DNMT1** | |  | |
| --- | --- | --- | --- | --- | --- |
| **Variable** | **Overall**  **N = 24^1^** | **Low**  **N = 10 (42%)^1^** | **High**  **N = 14 (58%)^1^** | ***p*-value^2^** | |
| **Gender** |  |  |  | >0.999 | |
| male | 12 (50.00%) | 5 (50.00%) | 7 (50.00%) |  |  |
| female | 12 (50.00%) | 5 (50.00%) | 7 (50.00%) |  |  |
| **Age** |  |  |  | >0.999 | |
| ≤60 | 9 (37.50%) | 4 (40.00%) | 5 (35.71%) |  |  |
| ＞60 | 15 (62.50%) | 6 (60.00%) | 9 (64.29%) |  |  |
| **Alcohol** |  |  |  | >0.999 | |
| No | 13 (54.17%) | 5 (50.00%) | 8 (57.14%) |  |  |
| Yes | 11 (45.83%) | 5 (50.00%) | 6 (42.86%) |  |  |
| **Smoking** |  |  |  | 0.697 | |
| No | 11 (45.83%) | 4 (40.00%) | 7 (50.00%) |  |  |
| Yes | 13 (54.17%) | 6 (60.00%) | 7 (50.00%) |  |  |
| **Diabetes** |  |  |  | 0.680 | |
| No | 12 (50.00%) | 4 (40.00%) | 8 (57.14%) |  |  |
| Yes | 12 (50.00%) | 6 (60.00%) | 6 (42.86%) |  |  |
| **CVD** |  |  |  | >0.999 | |
| No | 17 (70.83%) | 7 (70.00%) | 10 (71.43%) |  |  |
| Yes | 7 (29.17%) | 3 (30.00%) | 4 (28.57%) |  |  |
| **TNM** |  |  |  | 0.002 | |
| stage I | 6 (25.00%) | 6 (60.00%) | 0 (0.00%) |  |  |
| stage II | 12 (50.00%) | 4 (40.00%) | 8 (57.14%) |  |  |
| stage III | 6 (25.00%) | 0 (0.00%) | 6 (42.86%) |  |  |
| ^1^n (%) | | | | | |
| ^2^Pearson's Chi-squared test; Fisher's exact test; Fisher's Exact Test for Count Data with simulated p-value  (based on 2000 replicates) | | | | | |
