# Supplementary material for: DNMT1‐Induced Downregulation of CBX7 Inhibits ERK Phosphorylation and Promotes Pancreatic Ductal Adenocarcinoma Progression
Source: FASEB J. 2025 May 19;39(10):e70571. doi: 10.1096/fj.202402903R (PMC12087528; doi:10.1096/fj.202402903R)
Supplement: Supplementary file 3 — Table S2. [file FSB2-39-e70571-s005.docx]

| **Table S2. Lists of Antibody** | | | |
| --- | --- | --- | --- |
| **Antibody** | **Company** | **Cat. No.** | **Dilution (Application)** |
| anti-TUBULIN | Abclonal | A12289 | 1:2,000 (WB) |
| anti-DNMT1 | Abcam | ab18453 | 1:1,000 (WB, IHC) |
| anti-CBX7 | Abcam | ab21873 | 1:1,000 (WB, IHC) |
| anti-ERK | Abcam | ab184699 | 1:1,000 (WB) |
| anti-p-ERK | Abcam | ab229912 | 1:1,000 (WB) |
| anti-Histone H3 | CST | 4620S | 1:50(CHIP) |
| Normal Rabbit IgG | CST | 2729S | 1:50(CHIP) |
