# Supplementary material for: DNMT1‐Induced Downregulation of CBX7 Inhibits ERK Phosphorylation and Promotes Pancreatic Ductal Adenocarcinoma Progression
Source: FASEB J. 2025 May 19;39(10):e70571. doi: 10.1096/fj.202402903R (PMC12087528; doi:10.1096/fj.202402903R)
Supplement: Supplementary file 4 — Table S3. [file FSB2-39-e70571-s003.docx]

| **Table S3. Primers for qPCR** | | |
| --- | --- | --- |
|  | **Forward Primer** | **Reverse Primer** |
| GAPDH | AGATCCCTCCAAAATCAAGTGG | GGCAGAGATGATGACCCTTTT |
| DNMT1 | AAGTCCGATGGAGAGGCTAA | GACTCTTCCTGAGGTTTCCG |
| CBX7 | GCGTGCGGAAGGGTAAAGT | GCTTGGGTTTCGGACCTCTC |
