# Supplementary material for: DNMT1‐Induced Downregulation of CBX7 Inhibits ERK Phosphorylation and Promotes Pancreatic Ductal Adenocarcinoma Progression
Source: FASEB J. 2025 May 19;39(10):e70571. doi: 10.1096/fj.202402903R (PMC12087528; doi:10.1096/fj.202402903R)
Supplement: Supplementary file 5 — Table S4. [file FSB2-39-e70571-s002.docx]

| **Table S4. Primers for MS-PCR** | | |
| --- | --- | --- |
|  | **Forward Primer** | **Reverse Primer** |
| CBX7-M | TTTTGTAATTCGAGGGGTTGC | TAATCCTTAATACCCAAACGAACG |
| CBX7-U | TTTTTTTGTAATTTGAGGGGTTGT | CATAATCCTTAATACCCAAACAAACA |
